# Study on the defect reduction of metal folding on the formed tooth top with finishing roller in gear rolling process

**DOI:** 10.1038/s41598-023-31767-6

**Published:** 2023-03-22

**Authors:** Xiaobin Fu, Peng Chen, Jiankang Wang, Guangqing Liu, Xiaobao Ma

**Affiliations:** 1 College of Mechanical and Vehicle Engineering, Taiyuan University of Technology, Taiyuan, 030024 Shanxi People’s Republic of China; 2 Engineering Research Center of Advanced Metal Composites Forming Technology and Equipment, Ministry of Education, Taiyuan, 030024 Shanxi People’s Republic of China; 3 Shandong Product Quality Inspection Research Institute, Jinan, 250102 Shandong People’s Republic of China

**Keywords:** Engineering, Mathematics and computing

## Abstract

Cross rolling process is a new method to manufacture large-diameter gears, which has great advantages. While during the gear manufacturing process with cross rolling, due to the difference of deformation mechanism between the right and left formed tooth profiles, a tip is pulled at the tooth top of the workpiece, which severely affects the forming quality. To eliminate the occurred defect, the finishing roller is proposed and designed, the motion equation of the finishing roller is established and solved, the principle of the height increase of the formed tooth is obtained. And also a simplified finite element (FE) model with finishing roller and non-finishing roller are established in the DEFORM-3D software. The comparison of the simulation results between two situations is analyzed and can be concluded that with the finishing roller, the protrusions at both sides of the tooth top of the workpiece at each stage are flattened by the finishing roller, and the accumulation of the tooth top protrusions is not going to occur, which means no extrusion and finishing of the tooth top of the workpiece are required. In addition, the experiment with the finishing roller is carried out and the effectiveness of the finishing roller can be verified.

## Introduction

**Figure 1:**
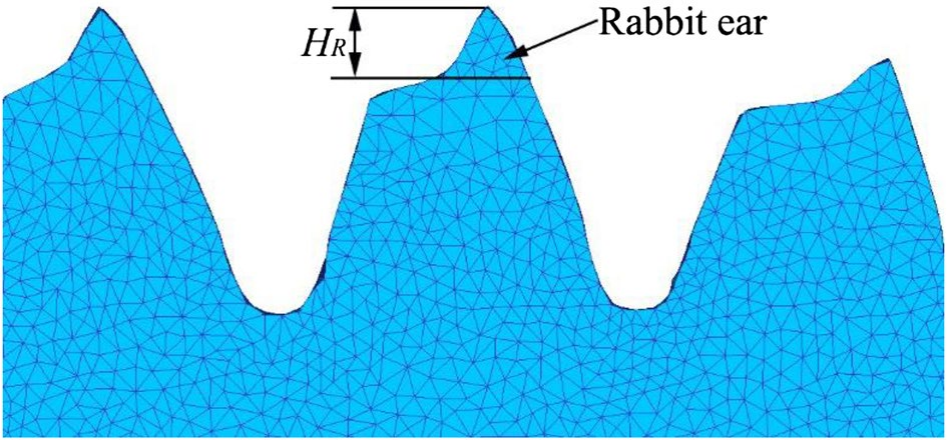
The rabbit ear on the tooth top of the formed workpiece^2^.

Gear rolling process is a new method to manufacture large-diameter gears, which has great advantages, such as higher production rates, considerable saving in metal, im-proved load capacity and extended tool life over conventional manufacturing methods^1^. However, during the process of gear manufacturing with gear rolling, due to the difference of deformation mechanism between the right and left tooth profiles, a tip is pulled at the tooth top, called rabbit ear, as Fig. 1 shows, which severely affects the forming quality, resulting the metal folding defect on the formed tooth top.^2^

To reduce the defect, many research works have been devoted. Kamouneh analyzed the defect of rabbit ear on the tooth top through the combination of finite element method and experiment and proposed a possible solution to reduce the defect. However, the possible solution was not verified by experiment^3–5^. Yu et al. studied forming of gear shaft by the cross wedge rolling process, and analyzed the influence of rolling temperature, friction force, workpiece shape and other changes on the phenomenon of rabbit ear and got a conclusion that the defect is caused by the upward flow of metal on the tooth profile caused by the friction between the die tooth and the formed tooth of the workpiece. While the measures to decrease the defect were not mentioned^6^. Wang studied the gear rolling forming by FE method, quantified the phenomenon of rabbit ear defect and studied the influence of different process parameters on metal folding on the tooth top. The height of rabbit ear was effectively reduced by preforming the workpiece and optimizing the process parameters. However, all the research data were based on finite element simulation, and none of experimental verification was carried out^7,8^. Zhu studied the influence of relative sliding between tooth profiles on metal flow during the rolling process, analyzed the factors which influence the defect of rabbit ear and summarized as that for forming gears with standard tooth height, increasing the forming temperature, and reducing friction are beneficial to reduce the defect of rabbit ear. After the gear die pressing for finish, the metal folding defect on tooth tip can be eliminated and carried out rolling experiments.^9^

**Figure 2:**
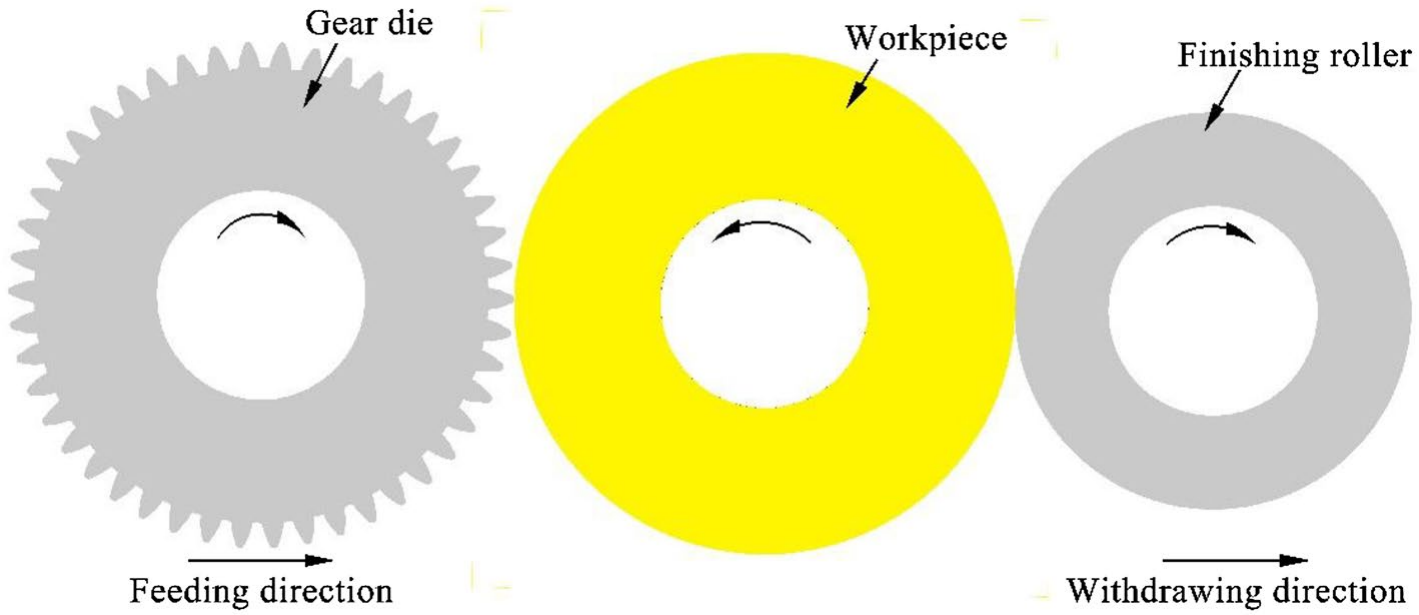
The principle of gear rolling process with finishing roller.

However, when forming gears with large modulus (greater than 5) or the high tooth gear, the process parameters optimization cannot be able to eliminate the defect of metal folding on the formed tooth top^10^. Therefore, in this paper a finishing roller device is designed. Shown as Fig. 2, during the gear rolling process, the tooth height of the workpiece is gradually increasing, the finishing roller rolls over the tooth top, and the metal pulled up by the friction is pressed to prevent the accumulation of the rabbit ear. Furthermore, the metal folding defect on tooth tip can be eliminated.

## FE model

As the formed tooth profile of the workpiece continues to grow, the roller needs to be retracted as Fig. 2 shows, so it is necessary to solve the motion equation of the finishing roller with the gear die feeding.

### The motion equations of finishing roller

**Figure 3:**
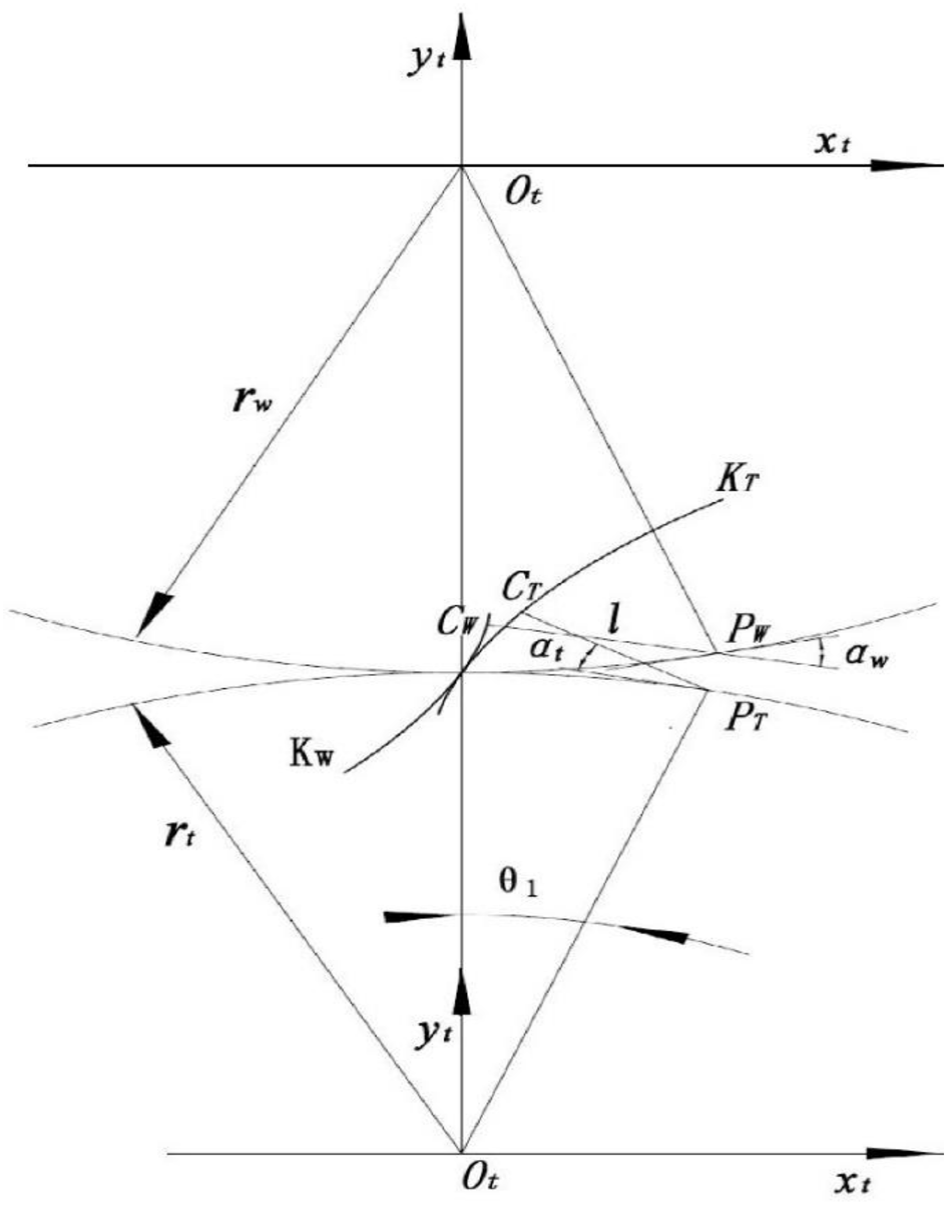
The meshing relationship of the involute tooth profile.

The motion equation of the finishing roller is established as follows, as Fig. 3 shows, *K*_*T*_ and *K*_*W*_ represent the conjugate tooth profile of the gear die and the workpiece respectively, and of those the radius of the dividing circle are *r*_*t*_ and *r*_*w*_ respectively. The rotating coordinate system (*x*_*t*_*O*_*t*_*y*_*t*_) and (*x*_*w*_*O*_*w*_*y*_*w*_) are respectively fixed with the gear die tooth profile *K*_*T*_ and the workpiece tooth profile *K*_*W*_. Moreover, the origin of the coordinate coincides with the rotation center of the workpiece and the gear die. At the beginning, the *y*_*t*_ axis and *y*_*w*_ axis coincide with the centerline of the workpiece and gear die through the node *P*. Assume that the normal line of a point *C*_*T*_ on the gear die tooth profile *K*_*T*_ intersects with the pitch circle in *P*_*T*_, and the normal line of the point *C*_*W*_ on the workpiece tooth profile *K*_*W*_ intersects with the pitch circle in *P*_*W*_, When the gear die tooth profile *K*_*T*_ turns counterclockwise to *θ*_*1*_, correspondingly the workpiece tooth profile *K*_*W*_ turns clockwise to *θ*_*2*_, *P*_*T*_ and *P*_*W*_ move to point *P* at the same time, and the points *C*_*T*_ and *C*_*W*_ begin to contact at *C* point. According to the meshing law, it can be obtained as follows,

1 $$ \overline{{C_{T} P_{T} }} = \overline{{C_{W} P_{W} }} $$

2 $$ PP_{T} = PP_{W} = r_{t} \theta_{1} = r_{w} \theta_{2} $$

3 $$ \alpha_{t} = \alpha_{w} $$

where, $\alpha_{t} ,\alpha_{w}$ are respectively the meshing angles of the tooth profile *K*_*T*_ and *K*_*W*_. For the convenience to analysis, let

4 $$ \alpha { = }\alpha_{t} = \alpha_{w} $$

5 $$ l = \overline{{C_{T} P_{T} }} = \overline{{C_{W} P_{W} }} $$

In here, define *l* as the length of the normal line.

The parameter equation of the gear die tooth profile *K*_*T*_ in coordinate system (*x*_*t*_*O*_*t*_*y*_*t*_)can be expressed as,

6 $$ \left\{ \begin{gathered} x_{t} = r_{t} \sin \theta_{1} - l\cos (\alpha + \theta_{1} ) \hfill \\ y_{t} = r_{t} \cos \theta_{1} + l\sin (\alpha + \theta_{1} ) \hfill \\ \end{gathered} \right. $$

The arc equation of the tooth top of the gear die in the coordinate system(*x*_*t*_*O*_*t*_*y*_*t*_)can be shown as follows,

7 $$ \left\{ \begin{gathered} x_{t} = r_{a} \sin \theta \hfill \\ y_{t} = r_{a} \cos \theta \hfill \\ \end{gathered} \right. $$

where, *θ* is the angle between the arc radius of die tooth top and *y*_*t*_ axis. The parameter equation of the workpiece tooth profile *K*_*W*_ can be expressed as,

8 $$ \left\{ \begin{gathered} x_{w} = r_{w} \sin \theta_{2} - l\cos (\alpha - \theta_{2} ) \hfill \\ y_{w} = - r_{w} \cos \theta_{2} + l\sin (\alpha - \theta_{2} ) \hfill \\ \end{gathered} \right. $$

and

9 $$ i = \frac{{\theta_{1} }}{{\theta_{2} }} = \frac{{r_{w} }}{{r_{t} }} = \frac{{\omega_{t} }}{{\omega_{w} }} $$

where, *i* is the transmission ratio for the gear die and workpiece, and since the rolling equipment adopts forced graduation, *i* is the fixed value.

As the normal line of the gear die tooth profile *K*_*T*_ at point *C*_*T*_ is always perpendicular to the tangent of that point, the angle between the normal at point *C*_*T*_ and the *x*_*t*_ axis is *π-(α* + *θ*_*1*_*)*, thus it can be obtained as,

10 $$ \frac{{\frac{{dy_{t} }}{{d\theta_{1} }}}}{{\frac{{dx_{t} }}{{d\theta_{1} }}}} = \cot (\alpha + \theta_{1} ) $$

According to Eq. (6), derivate for *θ*_*1*_ respectively, $\frac{{dy_{t} }}{{d\theta_{1} }}$ and $\frac{{dx_{t} }}{{d\theta_{1} }}$ can be obtained, substitute to Eq. (10), after simplified finishing, it can be obtained as

11 $$ \frac{dl}{{d\theta_{1} }} = r_{t} \cos \alpha $$

Since the meshing angle of the involute profile is constant, the Eq. (11) can be expressed as,

12 $$ l = r_{t} \theta_{1} \cos \alpha $$

Replace Eq. (12) with Eqs. (6) and (8) to obtain the gear die tooth profile equation as follows,

13 $$ \left\{ \begin{gathered} x_{t} = r_{t} \sin \theta_{1} - r_{t} \theta_{1} \cos \alpha \cos (\alpha + \theta_{1} ) \hfill \\ y_{t} = r_{t} \cos \theta_{1} + r_{t} \theta_{1} \cos \alpha \sin (\alpha + \theta_{1} ) \hfill \\ \end{gathered} \right. $$

And the workpiece tooth profile equation can be shown as

14 $$ \left\{ \begin{gathered} x_{w} = r_{w} \sin \theta_{2} - r_{t} \theta_{1} \cos \alpha \cos (\alpha - \theta_{2} ) \hfill \\ y_{w} = - r_{w} \cos \theta_{2} + r_{t} \theta_{1} \cos \alpha \sin (\alpha - \theta_{2} ) \hfill \\ \end{gathered} \right. $$

To facilitate the solution of the height increase of the formed tooth, the die tooth profile is rotated counterclockwise *φ*_*1*_. Accordingly, the workpiece tooth profile equation is rotated clockwise *φ*_*2*_*, φ*_*1*_ = *iφ*_*2*_, and it can be obtained as,

15 $$ \left\{ \begin{gathered} x_{t}^{^{\prime}} \hfill \\ y_{t}^{^{\prime}} \hfill \\ \end{gathered} \right\} = \left[ \begin{gathered} \cos \varphi_{1} \, - \sin \varphi_{1} \hfill \\ \sin \varphi_{1} \, \cos \varphi_{1} \hfill \\ \end{gathered} \right]\left\{ \begin{gathered} x_{t} \hfill \\ y_{t} \hfill \\ \end{gathered} \right\} $$

16 $$ \left\{ \begin{gathered} x_{w}^{^{\prime}} \hfill \\ y_{w}^{^{\prime}} \hfill \\ \end{gathered} \right\} = \left[ \begin{gathered} \cos \varphi_{2} \, \sin \varphi_{2} \hfill \\ - \sin \varphi_{2} \, \cos \varphi_{2} \hfill \\ \end{gathered} \right]\left\{ \begin{gathered} x_{w} \hfill \\ y_{w} \hfill \\ \end{gathered} \right\} $$

where, *φ*_*1*_ is the angle between the symmetric center line of the gear die tooth profile *K*_*T*_ and *y*_*t*_.

**Figure 4:**
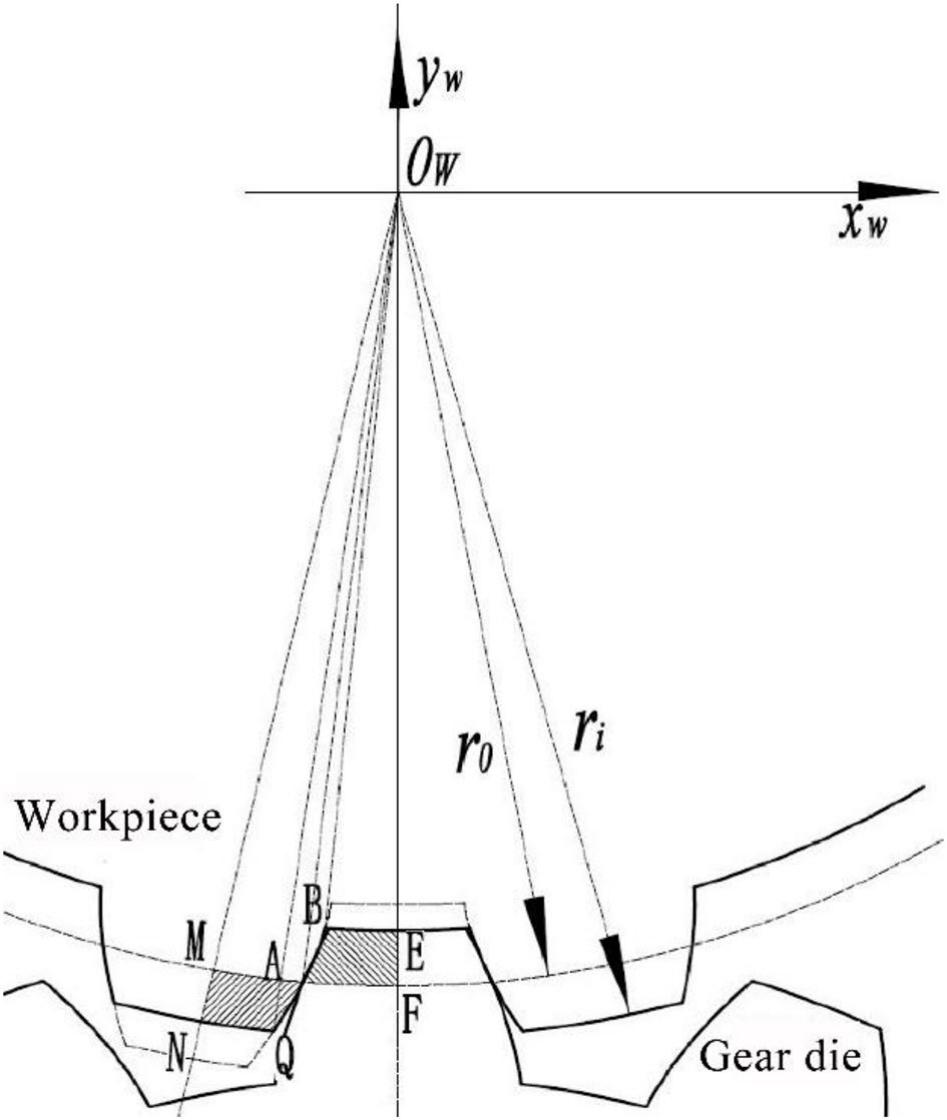
The schematic diagram of the position relationship between gear die and workpiece at a certain time.

The tooth profile after rotation is shown in Fig. 4 Point *A* is the intersection of the outer circle of the original workpiece and the involute. The initial radius of workpiece is *r*_*0*_, Point *Q* is the intersection point between the addendum circle of the workpiece tooth and the involute at certain time, and the radius is *r*_*i*_. *B* is the intersection of the tooth root circle and the involute of the formed part at a certain time.

Due to the principle of constant volume of the workpiece before and after forming in plastic processing, the generated area is equal to the area of the deep groove as *S*_*MNQA*_ = *S*_*BAEF*_, and it can be obtained as,

17 $$ S_{BAEF} = S_{AOF} - S_{BOE} - S_{AOB} $$

18 $$ S_{MNQA} = S_{{QO_{W} N}} - S_{{AO_{W} M}} - S_{{AO_{W} Q}} $$

According to the above theory, MATLAB software is used to numerically calculate the real-time diameter of the addendum circle of the workpiece in the forming process as a specific example, in which the basic parameters of the tooth of gear die and workpiece are shown in Table 1. The tooth height increase calculated by MATLAB at different times are shown in Table 2.

**Table 1:** The tooth shape parameters of gear die and formed workpiece.

Tooth shape parameters	Gear die	Workpiece
Tooth number (z)	112	74
Modulus (m)	3 mm	
Pressure angle (α/°)	17.5	
The addendum circle diameter	344.1 mm	230.9 mm
The root circle diameter	326.6 mm	213.4 mm

**Table 2:** The numerical calculation results of workpiece tooth height increase at different times.

Time *t (*s)	Feeding amount *f*_*1*_ (mm)	Tooth height increase h (mm)
0	0	0
5.3	0.53	0.02
10.5	1.02	0.06
15.9	1.57	0.084
21.2	2.09	0.1
26.6	2.62	0.15
32	3.17	0.22
37.4	3.71	0.43
42.7	4.51	0.79
48.1	4.78	1.02
53.4	5.32	1.3
58.7	5.85	1.61
64.1	6.35	2.03

The polynomial fitting is performed on the data of feeding amount and workpiece tooth height increase, which can obtain the function of *h* to *f*_*1*_, shown as,

19 $$ h(f_{1} ) = p_{1} + p_{2} f_{1} + p_{3} f_{1}^{2} + p_{4} f_{1}^{3} + p_{5} f_{1}^{4} + p_{6} f_{1}^{5} + p_{7} f_{1}^{6} $$

And the polynomial coefficient values are shown in Table 3.

**Table 3:** The polynomial fitting coefficient value of the relationship between feeding amount and the tooth height increase of workpiece.

Parameters	*p*_*1*_	*p*_*2*_	*p*_*3*_	*p*_*4*_	*p*_*5*_	*p*_*6*_	*p*_*7*_
Value	2.17E−3	− 0.147	0.471	− 0.374	0.127	− 1.85E−2	9.84E−4

Moreover, during the gear rolling process the feeding amount *f*_*1*_ = *vf*_*1*_*t*, where, *vf*_*1*_ is the feeding rate, thus the speed of the finishing roller *v*_*ya*_ can be obtained as

20 $$ v_{ya} (t) = \frac{{dh(f_{1} )}}{{df_{1} }}\frac{{df_{1} (t)}}{dt} = \, p_{2} + 2p_{3} (v_{{f_{1} }} t) + 3p_{4} (v_{{f_{1} }} t)^{2} + 4p_{5} (v_{{f_{1} }} t)^{3} + 5p_{6} (v_{{f_{1} }} t)^{4} + 6p_{7} (v_{{f_{1} }} t)^{5} $$

### FE model establishment and verification

**Figure 5:**
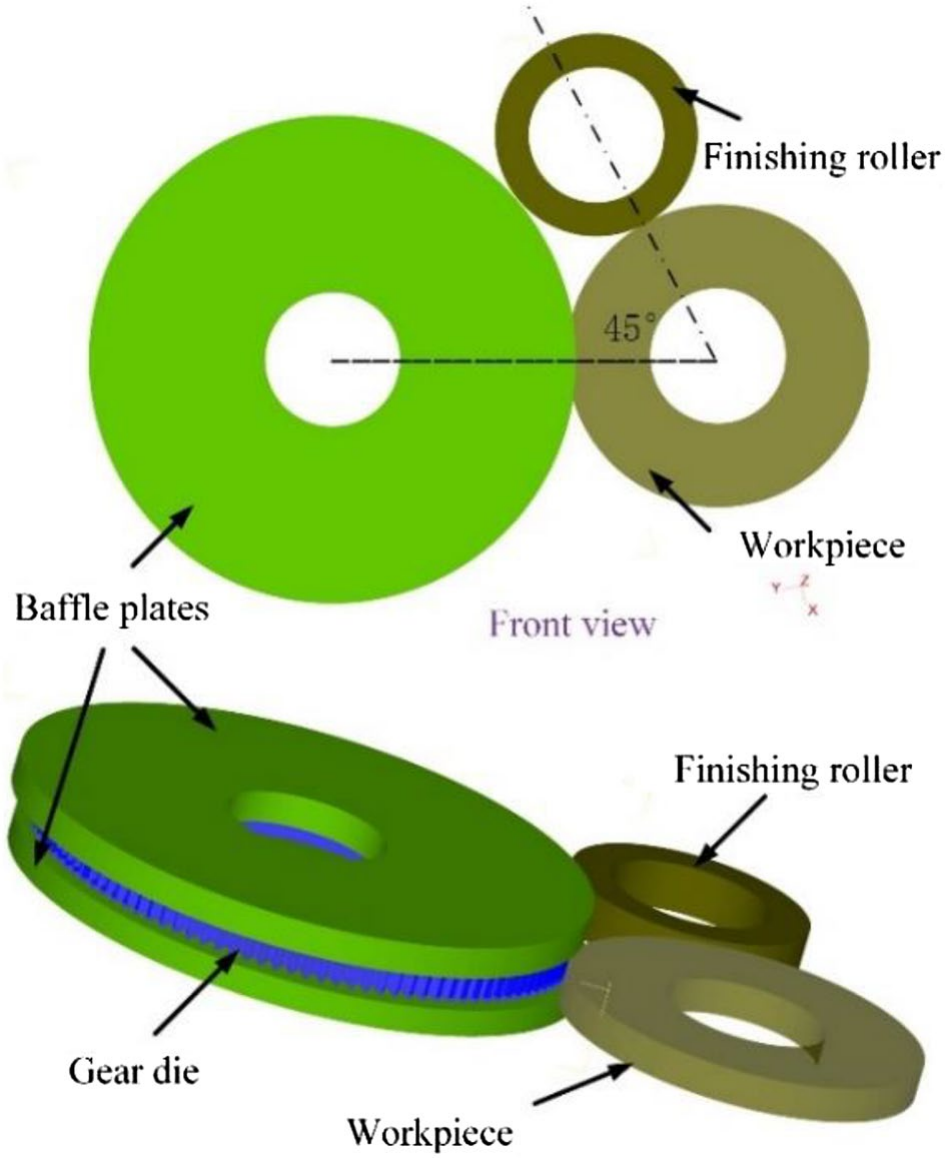
The FE model of gear rolling process with finishing roller.

The FE model of the gear rolling process with the finishing roller device is established and the assignment of the objects are shown in Fig. 5 considering the avoid the space interference. The workpiece is set as a rigid-plastic body and fixed. The gear die, the baffle plate and the finishing roller are set as rigid bodies. Since the workpiece is fixed, the gear die and the baffle plate rotate around their own axis, simultaneously rotating around the central axis of the workpiece. The finishing roller rotates around the central axis of workpiece too. And the rotation torque is set to 0, that is, when contacting with the workpiece, the finishing roller rotates driven by the friction from the workpiece. In addition, with the feeding motion, the gear die and the baffle plate gradually move close to the workpiece, and the finishing roller gradually moves away from the workpiece as the height of the formed tooth height of the workpiece increases. Since the gear die is 45° away from the finishing roller, the relationship between the speed of the finishing roller and time can be obtained as follows

21 $$ \begin{gathered} v_{ya} (t) = \left\{ \begin{gathered} \, 0 \, 0 \le t \le \pi /4\omega_{w} \hfill \\ v_{ya} (t - \pi /4\omega_{w} ) \, t \ge \pi /4\omega_{w} \hfill \\ \end{gathered} \right. \hfill \\ \hfill \\ \end{gathered} $$

where, *ω*_*w*_ is the speed of the workpiece during the rolling process (rad/s).

**Figure 6:**
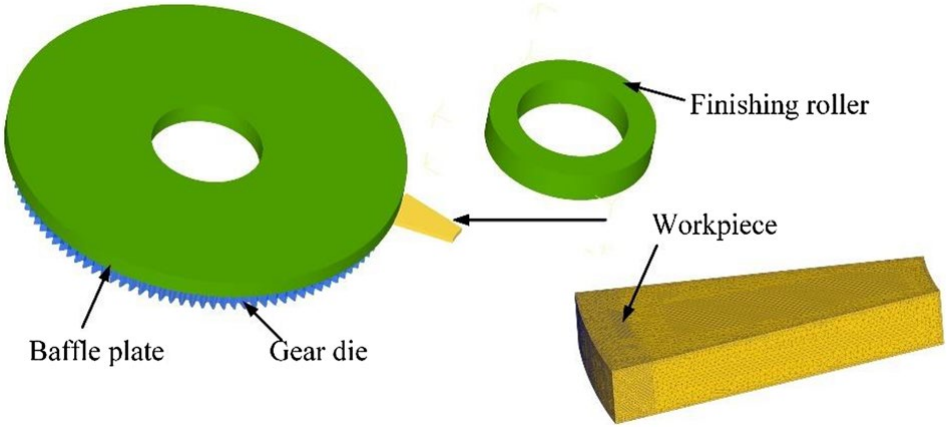
The simplified model of gear rolling process with finishing roller.

According to the characteristics of symmetry of the workpiece, half of the thickness of workpiece was taken for simplification^11,12^, shown in Fig. 6. In addition, since the gear die and workpiece are driven by their respective servo motors, both have certain speeds, and the parameters such as rolling force are periodically symmetrical, the workpiece model can be simplified to 1/12 fan shaped cylinder in the circumferential direction^13^. To improve the computational efficiency, the local mesh refinement of the outer surface forming area of the workpiece is carried out, which can ensure the accuracy of the involute shape of the formed tooth profile. At the same time, the time step is set to 0.01 s per step in the simulation process. As to the heat transmission, the ambient temperature is set as 20 °C, the contact heat transfer coefficient between gear blank and tooth mold is set as 5 × 10^3^ W/m^2^·K, the convection coefficient between gear blank and air is set as 20 W/m^2^·K, and the thermal radiation rate is 0.7. In addition, the shear friction model is selected for the process^14^. The friction force of the contact surface of the shear friction model does not change with the change of normal pressure and the unit friction force is constant, which conforms to the law of constant friction, which can be expressed as

22 $$ f = m \times k $$

where, *m* is the factor of friction, and *0* < *m* ≤ *1.0*, *k* is the shear yield stress of the workpiece.

The parameters of the finite element simulation of gear rolling process settings are shown in Table 4.

**Table 4:** The parameters of the finite element simulation settings.

Parameters	Value
Speed of gear die (rpm)	9.52
Speed of workpiece (rpm)	6.29
Feeding speed (mm/s)	0.15
Temperature of workpiece (°C)	1050
Temperature of gear die (°C)	20
Friction factor	0.3
Mesh number of workpiece	120,000
Material of gear die	H13
Material of workpiece	SAE 8620H

### Experimental verification

**Figure 7:**
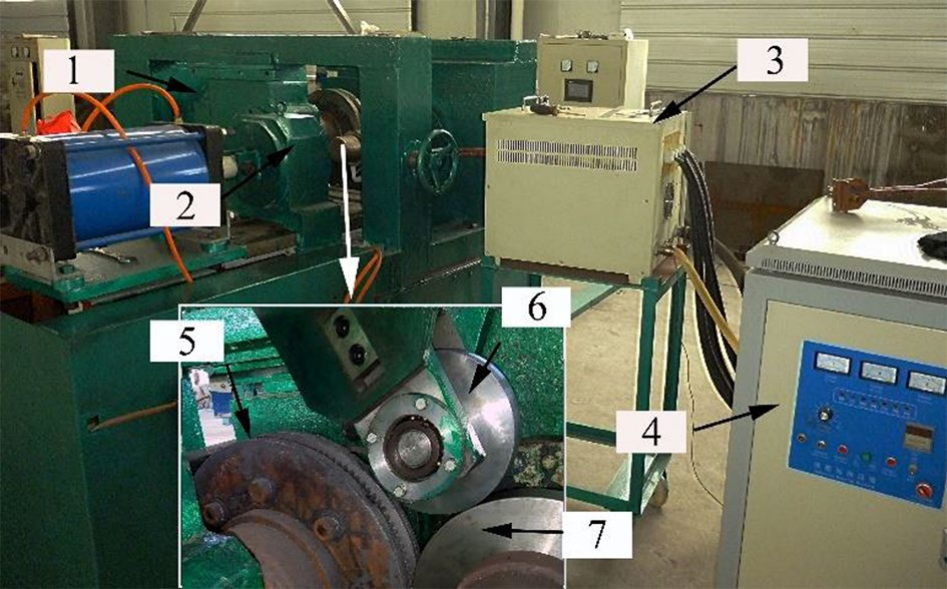
The equipment of gear rolling with finishing roller device. 1 Feeding device, 2 clamper, 3 transformer, 4 power source of induction heating and 5 gear die and baffles, 6 finishing roller, and 7 workpiece.

**Figure 8:**
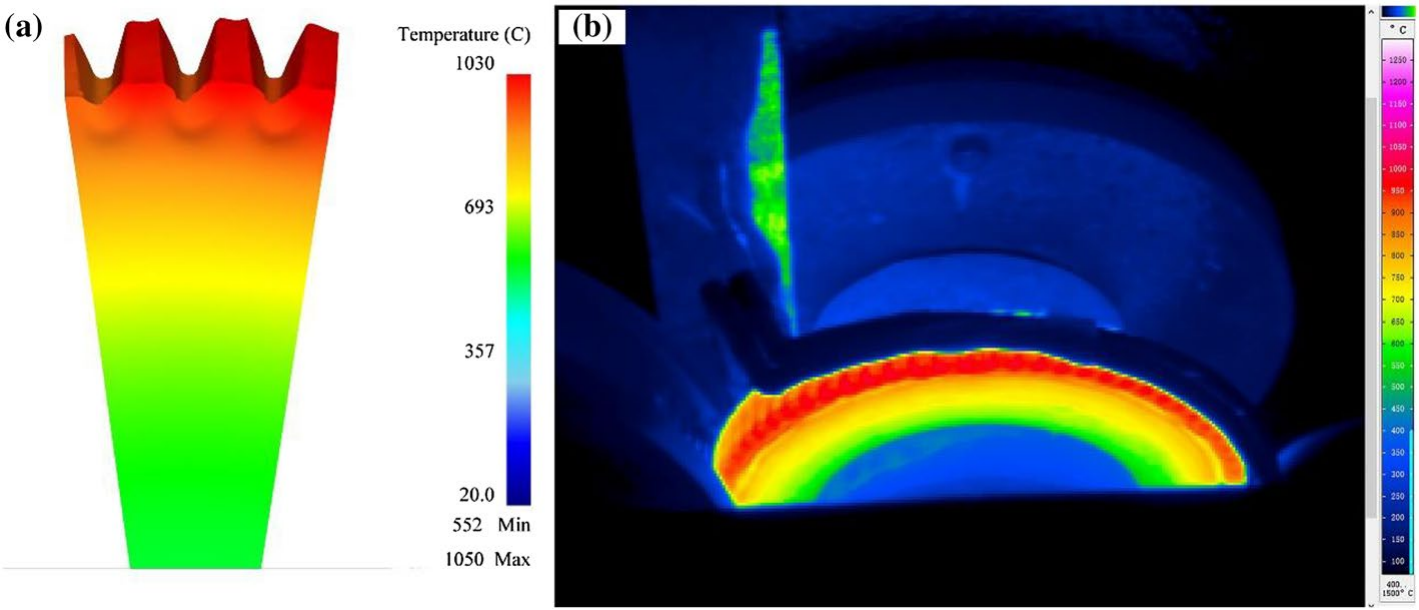
The temperature distribution of the workpiece in radial direction. (**a**) Simulation result and (**b**) experimental results captured by infrared thermographic imaging.

**Figure 9:**
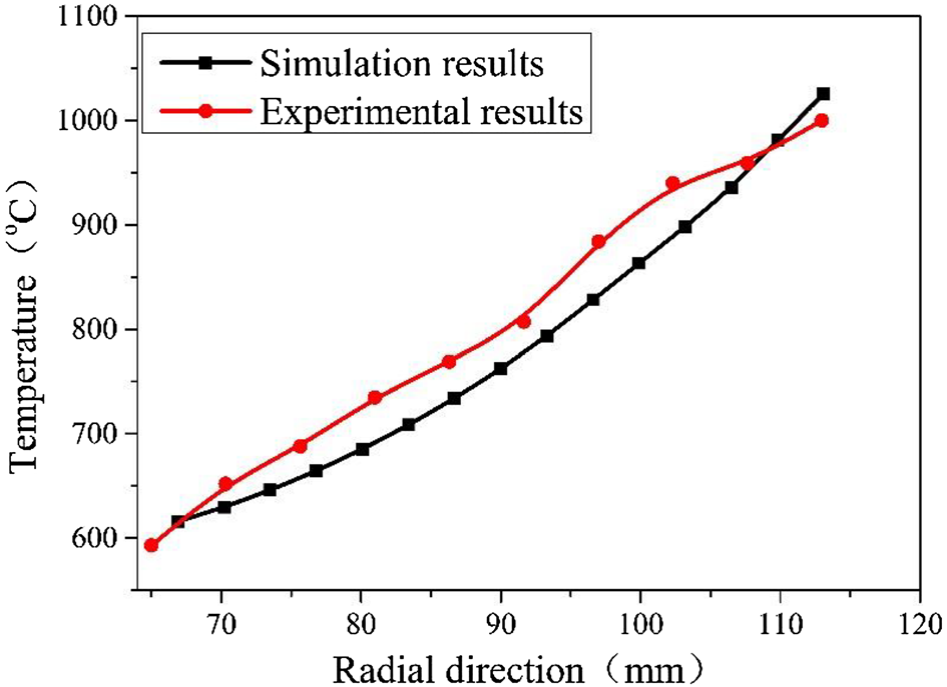
The quantified comparisons of temperature distribution of the formed workpiece.

**Figure 10:**
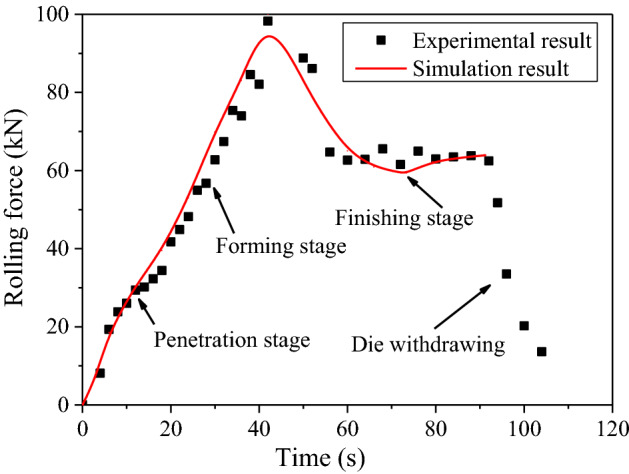
Comparison of simulation and experimental rolling force during different stages.

The gear rolling machine with finishing roller is shown in Fig. 7, and the gear rolling experiment is carried out. The comparisons of temperature distribution and rolling force between the experimental and simulation results during the gear rolling process was carried out. As shown in Fig. 8, the simulation result of the radial distribution of temperature has the same trends with experimental results. Moreover, the quantitative comparison is shown in Fig. 9 and near the forming zone, the maximum relative error of temperature distribution is about 3%. Therefore, the heating simulation results can be validated and considered reliable. As to the rolling force, shown in Fig. 10, at penetration and forming stage, the rolling force rises with the increase of feeding amount, then in finish stage, the rolling force drops and keep stable in certain value. According to the comparisons of the experimental and simulation results, the rolling force matches well, and the maximum relative error is 10.6%, which suggests the simulation results agree well with the experimental data, proving the validity of the established model^2^.

## Results and discussions

### Relationship between the feeding amount and the tooth height increase of workpiece

**Figure 11:**
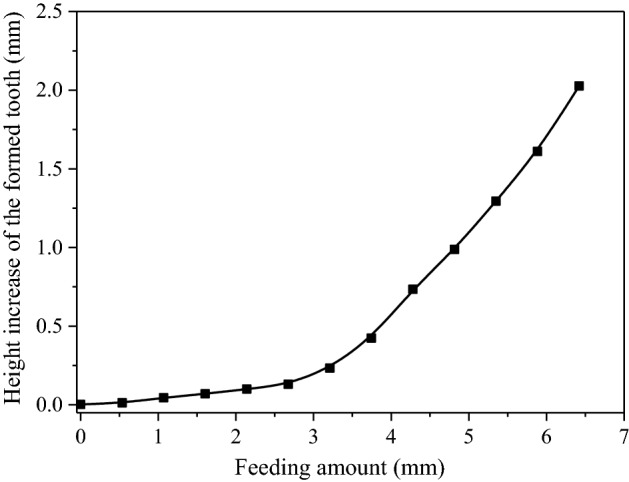
The relationship of the feeding amount and the height increase of the formed tooth.

According to Eq. (20), the relationship with the feeding and the tooth height increase of workpiece can be obtained and shown in Fig. 11, when the feed rate is small at the initial time, the height of formed tooth increases slow, which is because the tooth top of the die is thin and sharp. At this time, the radial flow metal extruded by the tooth top of the die is also less relatively. With the gradual increase of the feeding, the thickness of the gear die tooth profile penetrating the workpiece is also gradually increased, and the speed of the tooth height increase of workpiece is accelerated. As Fig. 11 shows, at the beginning of rolling, when the feeding amount increased from 0 to 0.55 mm, the workpiece tooth height increased by 0.02 mm, and when the feeding amount increased from 5.85 to 6.35 mm, the workpiece tooth height increased by 0.5 mm. In the later stage of the feed, a smaller feed rate can obtain a larger tooth height increase.

### Strain evolution and distribution

**Figure 12:**
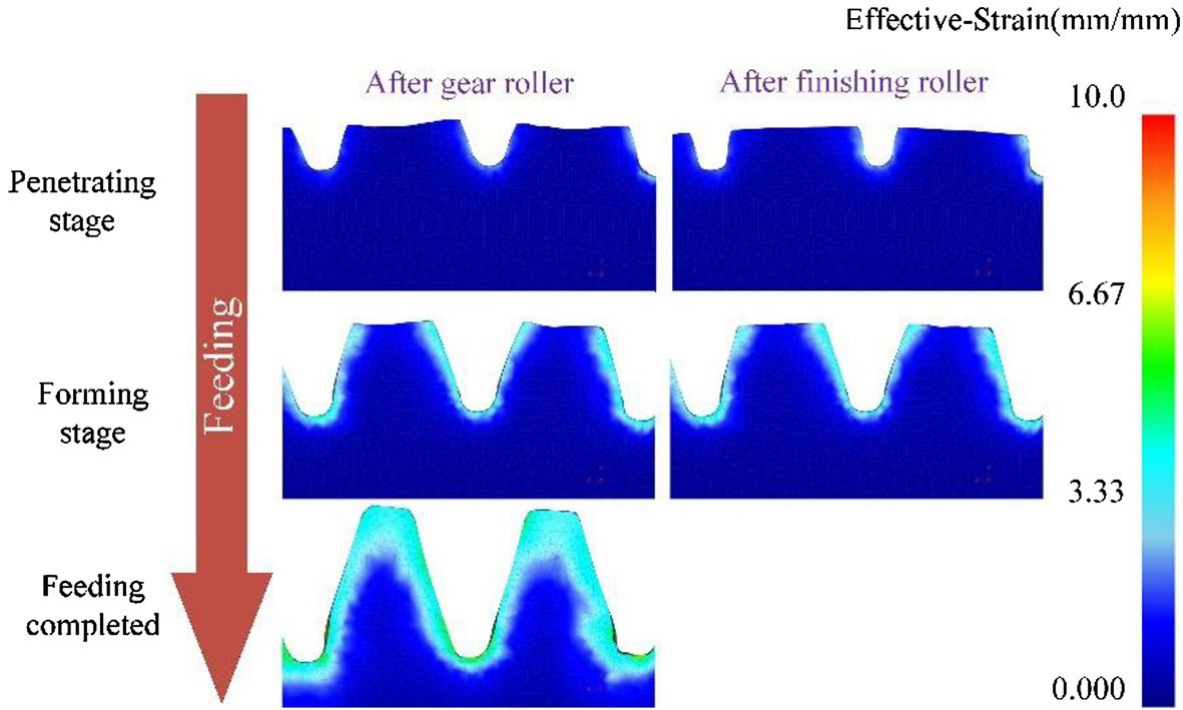
The strain contour of the formed tooth after gear rolling and finishing at certain pass.

The strain distribution of formed tooth at different stages of gear rolling process is shown in Fig. 12, at the penetrating stage, the effective strain of the deformed region in contact with the tooth profile of gear die, compared to that close to the heart of formed tooth, is greater and the strain close to the heart is almost 0, which suggests that in the rolling process, the deformation mainly occurs within a certain range from the surface of the workpiece. At the forming stage, compared with the former stage, the region with large effective strain becomes larger, mainly in the zone of the formed tooth root. With the gradual increase of feeding, the area with larger strain expands further. The effective strain changes gradually along the formed tooth width and drops to less than 0.75 at the center. Currently, there is a large difference in strain distribution between the two sides of the tooth profile, which indicates that the asymmetrical defect of the left and right profiles of the formed tooth occurs. When the feeding amount reaches 100%, the effective strain at the center of the formed tooth width increases indicating that the metal at the center also has extrusion deformation. Moreover, the asymmetry of the effective strain on both sides of the tooth profile is getting more serious. While the defect of the asymmetry of left and right tooth profile can be reduced by the finish process of alternating forward and reverse rotation, according to our former study^2^. As to the strain distribution after the finishing roller, compared with that after gear roller, strain contour changes at the tooth top, especially at two sides, caused by the squeezing of the finishing roller, resulting in the reduction of the defect of rabbit ear, and the strain at two sides of the tooth top get larger. When the feeding is finished, the defect of rabbit ear does not occur, the forming quality is great.

### The influence of the finishing roller device on the deformation of the tooth top of workpiece

**Figure 13:**
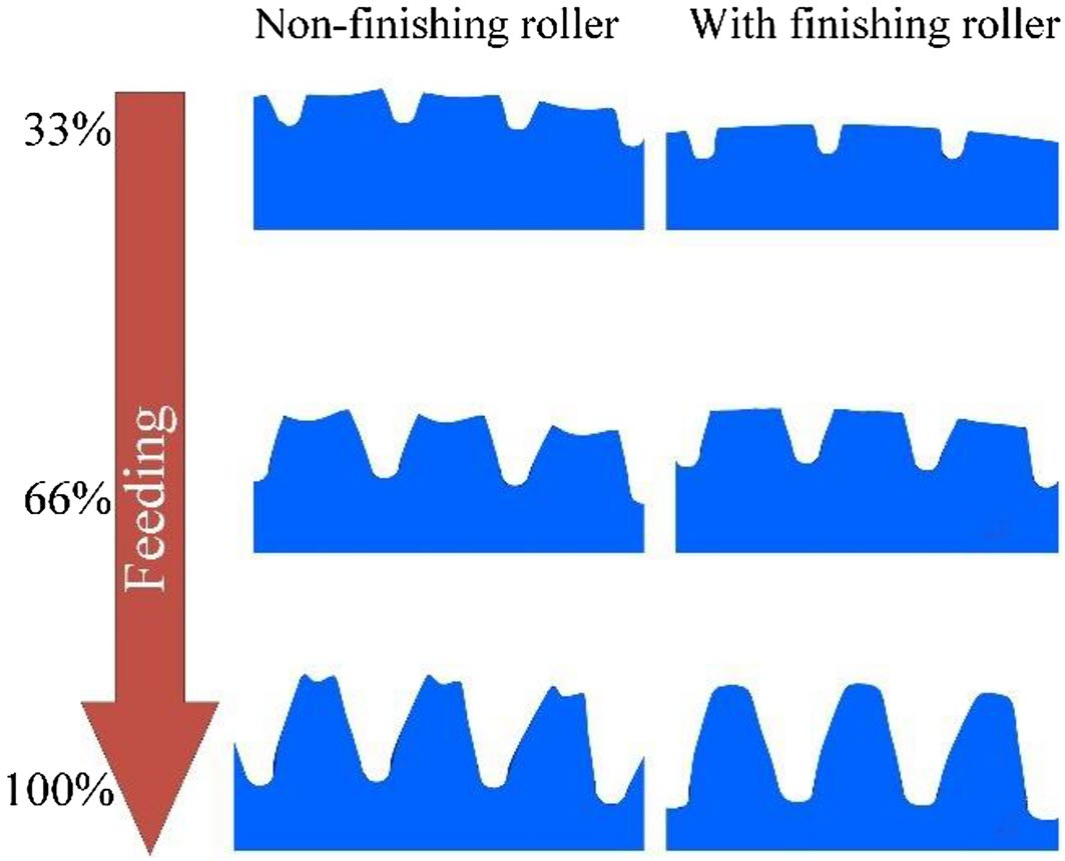
The comparisons of the forming results on two conditions, with finishing roller and the non-finishing roller.

**Figure 14:**
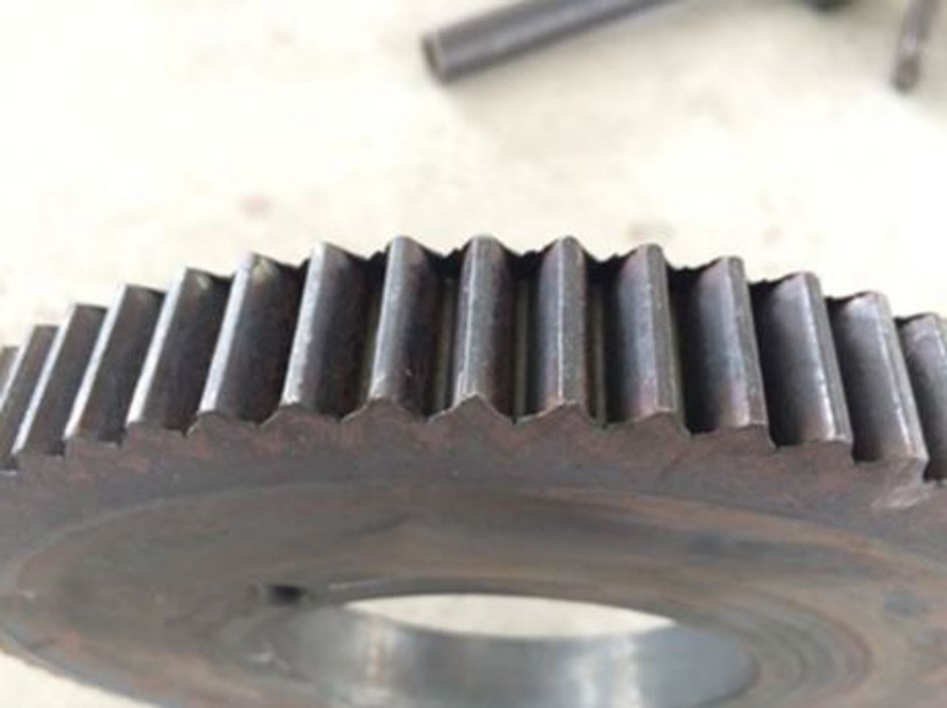
The formed tooth of the workpiece without metal folding defect using finishing roller device.

According to the finite element results, by comparing the forming results of two conditions, with finishing roller and the non-finishing roller, it can be figured out that in the initial stage of rolling, on the condition of non-finishing roller due to the friction caused by the relative sliding and the squeeze of the tooth space of the workpiece by the gear die tooth, the metal flow of the two sides of the profiles on the tooth top and the central area of the workpiece are different, resulting in the deformation of the depression at central area and the protrusion at the both sides on the tooth top of the workpiece. With the increase of feed, the tooth profile of the gear die penetrates the workpiece continuously, the tooth profile of the workpiece continues to grow, and the protrusions at both sides of the tooth top accumulates continuously until the end of the forming. The rabbit ears are formed at both sides of the tooth top of the workpiece. As shown in Fig. 13, when the feeding amount is 33%, the height of rabbit ear on the tooth top is 0.1 mm, and when the feeding amount is 66%, the height accumulates to 0.28 mm. Finally, when the forming is completed, the height accumulates to 0.59 mm. At this point, if the root of the tooth profile of the gear die continues to squeeze and finish the tooth top of the workpiece, the metal folding is going to occur. As to the condition of adding the finishing roller, the protrusions at both sides of the tooth top of the workpiece at each stage are flattened by the finishing roller, and the accumulation of the tooth top protrusions is not going to occur. With the continuous feeding of the gear die, the tooth profile of the workpiece is fully elongated, and there is no protrusion on the tooth top, which means no extrusion and finishing of the tooth top of the workpiece are required. There is no metal folding defect on the tooth top, and the forming quality is excellent. Moreover, the gear rolling experiment is carried out. The shape of the rolled tooth is shown in Fig. 14, which can be figured out that the tooth top is well formed. It can be verified that the metal folding defect on the tooth top can be effectively eliminated by adding the finishing roller device.

## Conclusions

In this paper, a new gear rolling process with designed finishing roller is proposed. The mathematical model of the motion relationship of the finishing roller during the gear forming process is established and solved by numerical calculation. With the motion model, the numerical simulation and relevant experimental research is carried out, and the metal folding on the tooth top of the formed tooth in gear rolling process with finishing roller are analyzed. The main conclusions of this paper are as follows:

1. During the gear forming process, at the initial time, the height of formed tooth increases slow, which is because the tooth top of the die is thin and sharp. With the gradual increase of the feeding, the thickness of the gear die tooth profile penetrating the workpiece is also gradually increased, and the speed of the tooth height increase of workpiece is accelerated.
2. According to the simulation results, with the finishing roller, the protrusions at both sides of the tooth top of the workpiece at each stage are flattened by the finishing roller, and the accumulation of the tooth top protrusions is not going to occur. With the continuous feeding of the gear die, the tooth profile of the workpiece is fully elongated, and there is no protrusion on the tooth top, which means no extrusion and finishing of the tooth top of the workpiece are required.
3. The gear rolling experiment with the finishing roller is carried out and can be verified the metal folding defect on the tooth top can be effectively eliminated by adding the finishing roller device.

## Data Availability

The datasets used and/or analysed during the current study available from the corresponding author on reasonable request.
